# Draft Genome Sequence of *Lactobacillus plantarum* CMPG5300, a Human Vaginal Isolate

**DOI:** 10.1128/genomeA.01149-14

**Published:** 2014-11-13

**Authors:** Shweta Malik, Roland J. Siezen, Bernadet Renckens, Mario Vaneechoutte, Jos Vanderleyden, Sarah Lebeer

**Affiliations:** a KU Leuven, Centre of Microbial and Plant Genetics, Kasteelpark Arenberg, Leuven, Belgium; b University of Antwerp, Department of Bioscience Engineering, Groenenborgerlaan, Antwerp, Belgium; c Radboud University Nijmegen Medical Centre, Centre for Molecular and Biomolecular Informatics (CMBI), Nijmegen, The Netherlands; d Microbial Bioinformatics, Ede, The Netherlands; e Ghent University, Laboratory of Bacteriology Research, Ghent, Belgium

## Abstract

The draft genome of a highly auto-aggregating *Lactobacillus plantarum* strain isolated from a human vagina is reported. The peculiar phenotype also provides an adhesive and co-aggregative potential with various pathogens, which could be of significance in the vaginal niche. Detailed genome analysis could aid in identifying the adhesins of the strain.

## GENOME ANNOUNCEMENT

*Lactobacillus plantarum* is a highly versatile species found in a variety of environmental niches such as in the gastrointestinal tract, and food products including dairy, meat, and vegetable fermentations (1, 2). There has been considerable interest in the probiotic potential of this species (1, 3, 4), yet it has been restricted to the intestinal isolates. Being a less commonly found species in the vaginal niche (5, 6), there are no reports on the genomes of vaginal *L. plantarum* isolates yet. Here, we report the draft genome of a vaginal *Lactobacillus plantarum* strain CMPG5300 with a remarkably high auto-aggregative capacity and adhesive ability to vaginal epithelial cell line VK2/E6E7 (7). The strong adhesive and auto-aggregative property of the strain is related to proteinaceous structures, as it can be abolished by proteinase treatment. A gene deletion mutant of the sortase-encoding gene of the strain lost the capacity to auto-aggregate and bind to mannose-rich conjugates of yeast cells, suggesting a crucial role for sortase-dependent proteins for these characteristics (7). Further insight into the genome of the strain may help the identification of putative adhesins imparting the exceptional features to the strain. This could particularly be of interest for targeted approaches that aim at evading pathogens at the vaginal front.

The genomic DNA of the strain CMPG5300 was isolated as described before (7) and whole-genome sequencing was performed using the 454 GS FLX+ sequencing platform (Genomics Core, KU Leuven). The reads were assembled into contigs using Roche GS De Novo Assembler software (version 2.5p1). The read coverage within the contigs was 50-100×. The minimum overlap length was set to 40 nucleotides, with a minimum overlap identity of 90%. Open-reading frames were extracted using the getorf program from the EMBOSS suite v6.3.1 (8), automatically annotated using the RAST server (9), and manually curated by comparison against the published genome of *L. plantarum* WCFS1 (10) using ACT (11).

The genome assembled into 48 contigs larger than 750 nt (containing a total 3,503,628 nt) of which at least six contigs represent plasmids (size 2 to 40 kb; total ~143 kb), with a total G+C content of 44%. The genome contains 3,251 protein-encoding genes (at least 140 are plasmid encoded). The scaffolded chromosome (19 contigs) is highly syntenous to published *L. plantarum* chromosomes (10, 12–16) (accession no. AL935263.2, CP001617.1, CP002222.1, CP004082.1, AGRI00000000.1, CP006033.1, PRJNA203333). Large variations are found between all *L. plantarum* chromosomes, including CMPG5300, in gene clusters encoding prophages, exopolysaccharide biosynthesis, plantaricin biosynthesis, and sugar metabolism (17). The putative plasmids of strain CMPG5300 encode a few sortase-dependent proteins, which may be relevant for the auto-aggregating and adhesive properties of this strain (7). These include a cell-envelope associated serine proteinase (Cmpg5300_3052) of 1,482 amino acids, which is 99% identical to plasmid-encoded proteinases of *L. plantarum* strains 16 and P8. Furthermore, a mucus-binding protein of 1,203 amino acids Cmpg5300.05_29 is encoded that is 98% identical to a cell-surface protein of *Lactobacillus antri*, and 62% identical to the mannose-specific adhesin (Msa) of *L. plantarum* WCFS1.

### Nucleotide sequence accession numbers.

This whole-genome shotgun project has been deposited at DDBJ/EMBL/GenBank under the accession no. AXZV00000000. The version described in this paper is version AXZV01000000.
